# Optimal Conditions for the Control Problem Associated to a Biomedical Process

**DOI:** 10.1063/1.3497987

**Published:** 2010-09-30

**Authors:** O. Bundău, A. Juratoni, A. Chevereşan

**Affiliations:** a “Politehnica” University of Timişoara, Department of Mathematics, P‐ţa. Victoriei, No. 2, 300004, Timişoara, Romania; b University of Medicine and Pharmacology of Timişoara, Str. Eftimie Murgu, No. 2, Timişoara, Romania; University of Peloponnese; University of Buckingham; Technological Educational Institution of Chalkis

## Abstract

This paper considers a mathematical model of infectious disease of SIS type. We will analyze the problem of minimizing the cost of diseases trough medical treatment. Mathematical modeling of this process leads to an optimal control problem with a finite horizon. The necessary conditions for optimality are given. Using the optimality conditions we prove the existence, uniqueness and stability of the steady state for a differential equations system.

## THE MODEL

The mathematical model proposed in this paper is the simplest model of an epidemic diseases. It is based on the a simplified version of what is called an SIS model as in [3]. We denote by $N(t)$ the total size of the population in period $t$. We suppose that the population will be divided up into two groups of people, those that have been infected by the disease and are infective, and those that are susceptible to being infected by the disease. We will label the number of those infected in moment $t$ as $I(t)$ and the number susceptible in moment $t$ as $S(t)$. Therefore we have

$$I(t)+S(t)=N(t). \tag{1}$$

The name SIS comes from two population groups Susceptible and Infected. Individuals go from being susceptible to a disease to being infected. And then they recover and again become susceptible. The number of individuals treated is denoted by $M(t)$.

The SIS model with stationary populations, normalized to one consists of the following equations

$$\begin{cases} I(t)+S(t)=N(t)=1 \\ \frac{dI(t)}{dt}=(\beta I(t))S(t)-\lambda(I(t)-M(t))-\delta M(t) \end{cases} \tag{2}$$

From the first equation of the system (2) we obtain $S(t)=1-I(t)$, and we have

$$\frac{dI(t)}{dt}=\beta I(t)(1-I(t))-\lambda(I(t)-M(t))-\delta M(t). \tag{3}$$

In medical terms $\beta$ is the transmission coefficient of the disease, $\lambda$ is the spontaneous rate of recovery for an untreated infected person and $\delta$ is the rate of recovery with treatment. Treatment is assumed effective, hence $\delta > \lambda$.

Infection imposes a cost of treatment. The cost of treatment is $C(M(t))$, where this is interpreted as total social cost of treatment and is assumed to exhibit increasing marginal cost. These costs include materials, facilities and labor used in administering treatment.

Our economic problem is to choose $M(t)$, such that to minimize the cost of treatment, on the finite time, given by

$$\int_0^T C(M(t))e^{-\rho t}dt \tag{4}$$

taking into account of the infection equation (3) and initial number of individuals infected, $I(0)=I_0$. The cost function $C: R_+ \to R_+$ is of class $C^2$ and satisfies $C(0)=0$, $C'(M)>0$, $C''(M)>0, \forall M \geq 0$, and the parameter $\rho>0$ is the time preference rate.

CP1281, *ICNAAM, Numerical Analysis and Applied Mathematics, International Conference 2010, Vol.II*
edited by T. E. Simos, G. Psihoyios, and Ch. Tsitouras
© 2010 American Institute of Physics 978-0-7354-0835-7/10/$30.00

## DETERMINATION OF OPTIMALITY CONDITIONS

The economic problem from the previous section leads us to the following mathematical optimization problem $P^*$**.**

**The problem** $P^*$**.** To determine $(I^*, M^*)$ which minimize the following functional

$$\int_0^T C(M(t))e^{-\rho t}dt \tag{5}$$

with $I \in AC([0,T],[0,1])$, $M \in \mathscr{X} = \{M : [0,T] \to [0,A], A \leq 1, M - measurable\}$, which verifies:

$$\begin{aligned} \dot{I}(t) &= \beta I(t)(1-I(t)) - \lambda(I(t)-M(t)) - \delta M(t) && (6)\\ I(0) &= I_0, && (7) \end{aligned}$$

where $AC([0,T],[0,1])$ is the class of absolutely continuous function. In our problem $P^*$**,** $I$ is the state variable and $M$ is a control variable.

In order to solve the minimum problem $P^*$ we will transform it into the maximum problem P.

**The problem P.** To determine $(I^*, M^*)$ which minimize the following functional

$$\int_0^T -C(M(t))e^{-\rho t}dt \tag{8}$$

with $I \in AC([0,T],[0,1])$, $M \in \mathscr{X} = \{M : [0,T] \to [0,A], A \leq 1, M - measurable\}$, which verifies:

$$\begin{aligned} \dot{I}(t) &= \beta I(t)(1-I(t)) - \lambda(I(t)-M(t)) - \delta M(t) && (9)\\ I(0) &= I_0, && (10) \end{aligned}$$

where $AC([0,T],[0,1]$ is the class of absolutely continuous function.

**Remark 1**. One can prove that the above problems are equivalent.

In what following we will determine the necessary conditions for optimal problem **P** as in [1], [2], [4]. To do this we will apply the principle of Pontryagin. For this we define the function of Hamilton-Pontryagin given by

$$H(I,M,\mu,t) = -C(M(t))e^{-\rho t} + \mu[\beta I(t)(1-I(t)) - \lambda(I(t)-M(t)) - \delta M(t)]. \tag{11}$$

**Theorem 2.** *Let* $(I^*(t), M^*(t))$ *be a optimal solution which solves problem* **P**. *Then there exists the adjoint absolutely continuous functions* $q(t)$ *such that for all* $t \in [0,T]$

$$\begin{aligned} q(t) &= \frac{C'(M^*(t))}{\lambda - \delta} && (12)\\ \dot{q}(t) &= q[2\beta I^*(t) + \lambda - \beta + \rho]. && (13) \end{aligned}$$

*Proof.* Let $(I^*(t), M^*(t))$ an optimal solution for **P**. The Hamilton function associated to the problem **P** is

$$H(I(t),M(t),\mu,t) = -C(M(t))e^{-\rho t} + \mu[\beta I(t)(1-I(t)) - \lambda(I(t)-M(t)) - \delta M(t)]. \tag{14}$$

From the Pontryagin's principle there exists the adjoint absolutely continuous function $\mu(t)$ such that

$$\dot{\mu}(t) = -\mu(t)(\beta - 2\beta I^*(t) - \lambda) \tag{15}$$

and $M^*(t)$ is value $M \in [0,A]$ which maximizes

$$H(I^*(t),M,\mu(t),t) = -C(M)e^{-\rho t} + \mu(t)[\beta I^*(t)(1-I^*(t)) - \lambda(I^*(t)-M) - \delta M]. \tag{16}$$

Using the transformation $\mu(t)(t) = e^{-\rho t}q(t)$, the Hamilton function becomes

$$H(I^*(t),M,q(t),t) = e^{-\rho t}\{-C(M) + q(t)[\beta I^*(t)(1-I^*(t)) - \lambda(I^*(t)-M) - \delta M]\}. \tag{17}$$

The first and second derivatives of function $H$ with respect to $M$ are

$$H'_M\left(I^*\left(t\right),M,q\left(t\right),t\right)=e^{-\rho t}[-C'(M)+q(t)(\lambda-\delta)] \tag{18}$$

$$H''_{MM}\left(I^*\left(t\right),M,q\left(t\right),t\right)=-e^{-\rho t}C''(M). \tag{19}$$

Using the property of the cost function we obtain that $H$ is a concave function of $M$.

Because $M^*(t)$ maximizes (17) and $H$ is a concave function of $M$ we have

$$H'_M\left(I^*\left(t\right),M^*(t),q\left(t\right),t\right)=0, \tag{20}$$

thus

$$q\left(t\right)=\frac{C'(M^*(t))}{\lambda-\delta}. \tag{21}$$

Using again the transformation $\mu\left(t\right)=e^{-\rho t}q\left(t\right)$, (15) becomes

$$\dot{q}\left(t\right)=q[2\beta I^*(t)+\lambda-\beta+\rho]. \tag{22}$$

**Definition 3.** A trajectory $(I(t),M(t))$ is called admissible for the problem **P** if it verifies the conditions (9), (10).
**Definition 4.** An admissible trajectory $(I^*(t),M^*(t))$, of the problem **P** is called optimal if

$$-\int_0^T e^{-\rho t}C(M(t))dt\leq-\int_0^T e^{-\rho t}(C(M^*(t))dt. \tag{23}$$

for every admissible trajectory $(I(t),M(t))$ of the problem **P**.

In next theorem, we give the sufficient conditions for the solution of our optimal control problem **P**.

**Theorem 5.** Let $(I^*(t),M^*(t))$ be an admissible trajectory in problem **P**. If there exists an absolutely continuous function $q(t)$ such that for all $t$, the following conditions are satisfied

$$q\left(t\right)=\frac{C'(M^*(t))}{\lambda-\delta}$$

$$\dot{q}\left(t\right)=q[2\beta I^*(t)+\lambda-\beta+\rho]$$

and the transversality condition

$$q(T)=0$$

holds, then $(I^*(t),M^*(t))$ is an optimal trajectory for the problem $P$.
*Proof.* Let $(I^*(t),M^*(t))$ be an arbitrary admissible trajectory for **P**. We denote

$$\Delta=\int_0^T -e^{-\rho t}C(M^*(t))dt-\int_0^T -e^{-\rho t}C(M(t))dt. \tag{24}$$

In the following, we simplify our notations and put $I^*(t)=I^*$, $M^*(t)=M^*$, $I(t)=I$, $M(t)=M$, $H(I,M,\mu,t)=H$, $H(I^*,M^*,\mu,t)=H^*$. Using the hamiltonian we have

$$-C(M)e^{-\rho t}=H(I,M,\mu,t)-\mu[\beta I(t)(1-I(t))-\lambda(I(t)-M(t))-\delta M(t)]. \tag{25}$$

By (9), (25) the relation (24) becomes

$$\Delta=\int_0^T[H(I^*,M^*,\mu,t)-H(I,M,\mu,t)]dt+\int_0^T\mu(\dot{I}-\dot{I}^*). \tag{26}$$

Since $H(I,M,\mu,t)$ is concave as a function of $I$ and $M$, using a standard result on concave functions, we obtain

$$H(I,M,\mu,t)-H(I^*,M^*,\mu,t)\leq\frac{\partial H^*}{\partial I}(I-I^*)+\frac{\partial H^*}{\partial M}(M-M^*). \tag{27}$$

Using the transformation $q(t) = e^{\rho t}\mu(t)$ in (13) we have the equation of the adjoint variable $\mu$ , $\frac{\partial H^*}{\partial I} = -\dot{\mu}$. Again, from (10), (27) and $\frac{\partial H^*}{\partial I} = -\dot{\mu}$ in (26), we obtain that

$$\Delta \geq \int_0^T [\dot{\mu}(I - I^*) + \mu(\dot{I} - \dot{I}^*)]dt + \int_0^T \frac{\partial H^*}{\partial M}(M^* - M)dt. \tag{28}$$

We differentiate (25) with respect to $M$ and using (12) we have the maximum condition

$$H(I^*(t), M^*(t), \mu(t), t) \geq H(I^*(t), M, \mu(t), t),$$

i.e.

$$\frac{\partial H^*}{\partial M}(M^* - M) \geq 0, \quad M \in [0, A] \tag{29}$$

From (29) we have that the second integral in (28) is positive, therefore

$$\Delta \geq \int_0^T [\dot{\mu}(I - I^*) + \mu(\dot{I} - \dot{I}^*)]dt \quad \textit{or equivalently}, \quad \Delta \geq \int_0^T \frac{d}{dt}[\mu(I - I^*)]dt = \mu(I - I^*)|_0^T = 0,$$

due to the initial and transversality conditions. Hence, we obtain that $(I^*(t), M^*(t))$ is an optimal trajectory for the problem **P**.

**Remark 6.** If we differentiate the condition (12) with respect to $t$ and we use (13) we obtain

$$\dot{M}(t) = \frac{C'(M(t))}{C''(M(t))}[2\beta I(t) + \lambda - \beta + \rho] \tag{30}$$

**Remark 7.** The optimal trajectory of the problem **P** is a solution of the following system of differential equations

$$\dot{I}(t) = \beta I(t)(1 - I(t)) - \lambda(I(t) - M(t)) - \delta M(t) \tag{31}$$

$$\dot{M}(t) = \frac{C'(M(t))}{C''(M(t))}[2\beta I(t) + \lambda - \beta + \rho] \tag{32}$$

**Proposition 8.** (Stationary state). *The nonlinear differential equations system (31)-(32) has a unique steady state* $(I^*, M^*)$, *given by*

$$I^* = \frac{\beta - \lambda - \rho}{2\beta}, \quad M^* = \frac{\rho^2 - (\beta - \lambda)^2}{4\beta(\lambda - \delta)}.$$

*Proof.* To determinate the steady state of the above system we choose the stationary solutions $I(t) = I^*$, $M(t) = M^*$.

From $\dot{M}(t) = 0$ we obtain $I^* = \frac{\beta - \lambda - \rho}{2\beta}$. Using $I^*$ in equation $\dot{I}(t) = 0$, we obtain $M^* = \frac{\rho^2 - (\beta - \lambda)^2}{4\beta(\lambda - \delta)}$.

## CONCLUSION

In this paper, we have analyzed a medical management model, where we minimize the cost of diseases trough medical treatment. We have determined the necessary and sufficient conditions for optimality. Using these conditions, we have shown that the optimal trajectory for the optimal control problem is the solution for a differential equations system. We have proven the existence and uniqueness of the study state.
